# Supplementary material for: Reproducible single-cell annotation of programs underlying T cell subsets, activation states and functions
Source: Nat Methods. 2025 Sep 3;22(9):1964–80. doi: 10.1038/s41592-025-02793-1 (PMC12446064; doi:10.1038/s41592-025-02793-1)
Supplement: Supplementary file 1 — Supplementary Note and Supplementary Figs. 1–10. [file 41592_2025_2793_MOESM1_ESM.pdf]

nature methods

Supplementary information https://doi.org/10.1038/s41592-025-02793-1

Reproducible single-cell annotation of programs underlying T cell subsets, activation states and functions

In the format provided by the authors and unedited

Supplementary Note

Labeling cGEPs using gene sets

We compared cGEPs with gene-sets from the gene ontology database[1] and from T cell polarization experiments[2] (**Methods, Supplementary Table 3**). We found that the Th2-Resting and Th2-Activated cGEPs were the most significantly enriched for genes upregulated following 16 hour stimulations of naive T cells with Th2 polarizing cytokines (Fisher Exact Test OR=22.7, 16.2, $P=4.9\times10^{-5}$, 1.7×10^{-4}, respectively). Gene set analysis also helped annotate 5 cGEPs corresponding to non-T cell specific cellular functions: early and late cell cycle S-phase ($P=3\times10^{-56}$ for DNA_REPLICATION and $P=2\times10^{-55}$ for MITOTIC_CELL_CYCLE), G2M-phase ($P=9\times10^{-74}$ CELL DIVISION), interferon stimulated genes ($P=1\times10^{-59}$ for RESPONSE TO VIRUS), and translation ($P=4\times10^{-163}$ for GOCC_CYTOSOLIC_RIBOSOME).

Description of immediate early gene (IEG) gene sets

We labeled three correlated cGEPs as immediate early gene programs (IEG, IEG2, IEG3, pairwise R of 0.45-0.70). The top genes include the canonical IEGs including *FOS*, *JUN*, and *ZFP36,* and these cGEPs were all enriched for a published IEG gene set[3] (Fisher Exact Test $P<1\times10^{-53}$). We suspect that IEG represents the core pathway as it was found in 6 out of 7 datasets (**Fig. 1c**) whereas IEG2 and IEG3 represent mixtures with delayed immediate and secondary response genes. We hypothesize that these cGEPs reflect sample processing artifacts in scRNA-Seq, since IEGs are induced in as few as 30 minutes[4] in response to mitogens or cell stress[5], and following processing steps like tissue disaggregation[6,7]. As evidence of the potential technical nature of these cGEPs, we calculated their mean usage per sample in T cells, B-cells, NK-cells and monocytes/DCs in the 3 PBMC references. We found that their average usage in T cells correlates with their usage in other cell-types (R=0.46-0.99, average

0.77, **Supplementary Fig. 4**), suggesting that they are a sample-intrinsic property, which would be expected of a sample-processing effect. However, in certain contexts, these cGEPs may be biologically important.

Clustering misclassified T cell subsets

In the COMBAT dataset, many CD4 memory subclusters were enriched for functional or artifact-associated cGEPs rather than subset cGEPs (**Supplementary Fig. 6b**). The CD4.Th.mitohi and CD4.Tem.mitohi.1 clusters were driven by high usage of the Poor-Quality cGEP and contained cells expressing multiple subset cGEPs. The CD4.TEM.IFN.resp and CD4.Th.IFN.resp clusters were predominantly driven by the ISG cGEP. The CD4.TEM.IFN.resp cluster had high usage of the cytotoxic and TEMRA cGEPs while the CD4.Th.IFN.resp cluster contained cells expressing many subset cGEPs including CD4-Naive (**Fig. 3b, Extended Data Fig. 4b**). Cells with high usage of the CD4-Naive cGEP expressed CD4 naive markers including CD45RA and *SELL*, confirming that clustering misclassified them as memory T cells (**Supplementary Fig. 6c-e**).

Description of cGEPs associated with antigen-specific activation in the AIM-Seq assay

Many suspected functional AIM-associated cGEPs included well known markers of T cell activation in their top weighted genes. We provide a brief description of these cGEPs below:

- CTLA4/CD38 showed the most upregulation in Tregs and CD4 memory cells (**Fig. 4F**) and is characterized by the CD278 and CD38 activation markers and the anti-

inflammatory genes *CTLA4* and *IL10*. It may thus reflect an inhibitory activation response.

- ICOS/CD38 included the known T cell activation markers CD278, CD71, and CD38 and showed the most upregulation with activation in conventional CD4 and CD8 T cells.
- OX40/EBI3 was identified specifically in tissues and was most common in tumor-infiltrating T-cells (Figure 1B, Supplementary Figure 7c-d). It is the only of these functional AIM-associated cGEPs to include *CXCL13*, a marker of tumor-reactivity and a B cell chemoattractant (**Supplementary Fig. 7c**). It also includes other activation markers such as *TNFRSF4* (encoding OX40) and *IL2RA* (encoding CD25).
- TIMD4/TIM3 is most expressed in MAIT, gdT, and CD8 memory T-cells and is characterized by expression of CD38 protein and RNA and cytotoxicity genes (*GZMB*, *GZMA*, *GNLY*), likely representing a cytotoxic activation response.
- NME1/FABP5 was broadly upregulated across all lineages. It is most strongly marked by expression of the *NME1* endonuclease encoded gene that was previously associated with T cell activation[8].
- Multi-cytokine was most strongly upregulated in CD8 memory cells and is most strongly marked by cytokine and chemokine encoding genes such as *IFNG*, *TNF*, *XCL1*, *IL2*, *CCL4*, *CCL4L2*, and *CCL3*.
- Th17-Activated is strongly marked by *IL26*, *IL17A*, *IL17F*, and *IL22* expression, encoding cytokines secreted by activated Th17 cells.

- Th2-Activated is marked by expression of *IL5*, *IL13*, and *IL4*, encoding cytokines secreted by activated Th2 cells.

Analysis of cGEPs associated with Covid-19

We tested cGEP associations with Covid-19 in two PBMC-derived T cell datasets, UK-Covid (80 Covid-19, 21 healthy donors, **Extended Data Fig. 7c**) and COMBAT (77 Covid-19, 10 healthy donors, **Extended Data Fig. 7d**), using psuedobulk sample-level regression (**Methods).** We observed overall concordant cGEP associations (Pearson R=0.64, $P=2.8\times10^{-7}$, **Extended Data Fig. 7e**). Consistent with the key role of interferon in viral infections[8,9], ISG was the most upregulated cGEP in both datasets (Q<0.05). AIM-associated functional cGEPs including exhaustion, cell cycle, TIMD4/TIM3, OX40/EBI3, NME1/FABP5, and CTLA4/CD38 were upregulated in acute Covid-19, consistent with viral activation of T cells (Q<0.05, both datasets). We also found increased Tph cGEP usage in Covid-19 relative to controls, consistent with recent demonstration of increased abundance of this subset in infection ($Q<1\times10^{-8}$, both datasets)[10]. An intriguing novel finding is that the Th1-like cGEP was negatively associated with Covid-19 in both datasets ($Q<1\times10^{-4}$). This was seen within manually gated CD4 CM ($Q=1.1\times10^{-4}$) and CD4 EM subsets ($Q=4.5\times10^{-6}$), suggesting it is not due to differential abundance of circulating memory CD4 T cells. Immediate early gene cGEPs (IEG1, IEG2, IEG3) were also significantly associated with Covid-19 in COMBAT ($Q<1\times10^{-5}$) but not UK-Covid (P>0.5), perhaps related to sample processing differences (see prior section on IEG cGEPs).

Analysis of cGEPs associated with RA

Next, we identified cGEPs associated with inflamed synovial tissue in rheumatoid arthritis (RA) using the AMP-RA dataset, which includes synovial biopsies from 70 RA and 8 osteoarthritis (OA) patients (**Extended Data Fig. 7f**)[11]. Ten out of the eleven significantly associated cGEPs were AIM-associated, including metallothionein, ISG, Tph, HLA, ICOS/CD38, Exhaustion, and all

three cell cycle cGEPs (Q<0.05 all). Of note, Metallothionein was shown to be increased in the plasma of RA patients and within the synovia of mouse models of RA[12]. The Tph association is consistent with prior observations by us and others of Tph enrichment within RA synovia[13]. The Th22 cGEP was also associated with RA (Q=0.0027), confirming a prior observation of increased Th22 cell abundance in RA synovia, where they may stimulate osteoclasts[14].

Analysis of Tph usage and plasma cells in cancer

Within the primary pan-cancer dataset, we found that the average Tph usage per sample was correlated with the expression of the B-cell chemoattractant *CXCL13* (R=0.67, P=1.2×10^{-30}, **Supplementary Fig. 9c**). This correlation was stronger in tumor (R=0.69, P=1.2×10^{-13}) than normal samples (R=0.34, P=0.021). We hypothesized that average Tph usage would correlate with plasma cell abundance in tumors. To test this, we re-analyzed a published pan-cancer dataset containing other cell-types besides T cells from 148 primary tumors, 53 matched adjacent tissues, and 25 healthy donor samples[15]. Tph usage and *CXCL13* expression remained correlated in this dataset (R=0.67, P=1.2×10^{-30}, **Supplementary Fig. 9d**). Average Tph, Tfh-1, and Tfh-2 cGEP usage were significantly correlated with plasma cell percentage within the tumors (Spearman ρ=0.23, 0.34, 0.28, respectively, P<1×10^{-2}, **Supplementary Fig. 9e**). In a multivariate regression across all samples, Tfh-1 and Tph usage were independently associated with plasma cell abundance (P=0.042, P=0.051 respectively). Subsetting to non-tumor samples, Tfh-1 and Tfh-2 remained statistically significant (P=0.017, P=0.027, respectively), but Tph was no longer significant (P=0.351). These findings suggest that Tph cells are functional within tumors and are associated with increased abundance of plasma cells.

Supplementary Figures

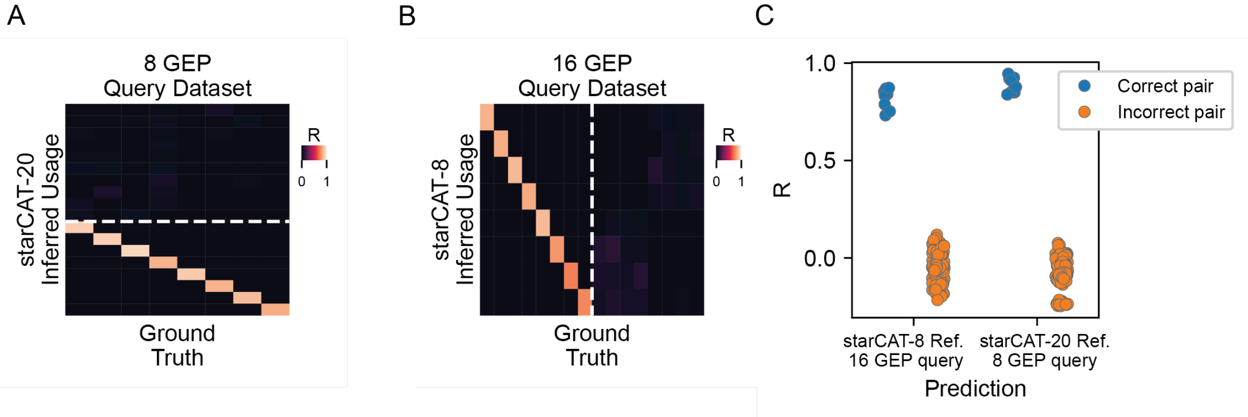

Supplementary Figure 1. Additional starCAT simulation analyses. a, Pearson correlation of ground truth simulated usages of each GEP (columns) vs inferred usages (rows) for starCAT using a simulated 20 GEP reference to predict an 8 GEP query dataset, where the 8 GEPs overlap with the reference. **b,** Same as A but using an 8 GEP reference and a 16 GEP query dataset, containing 8 overlapping GEPs. **c,** Extracted Pearson correlation values from **a** and **b** where dot color indicates whether an inferred GEP matched the ground truth.

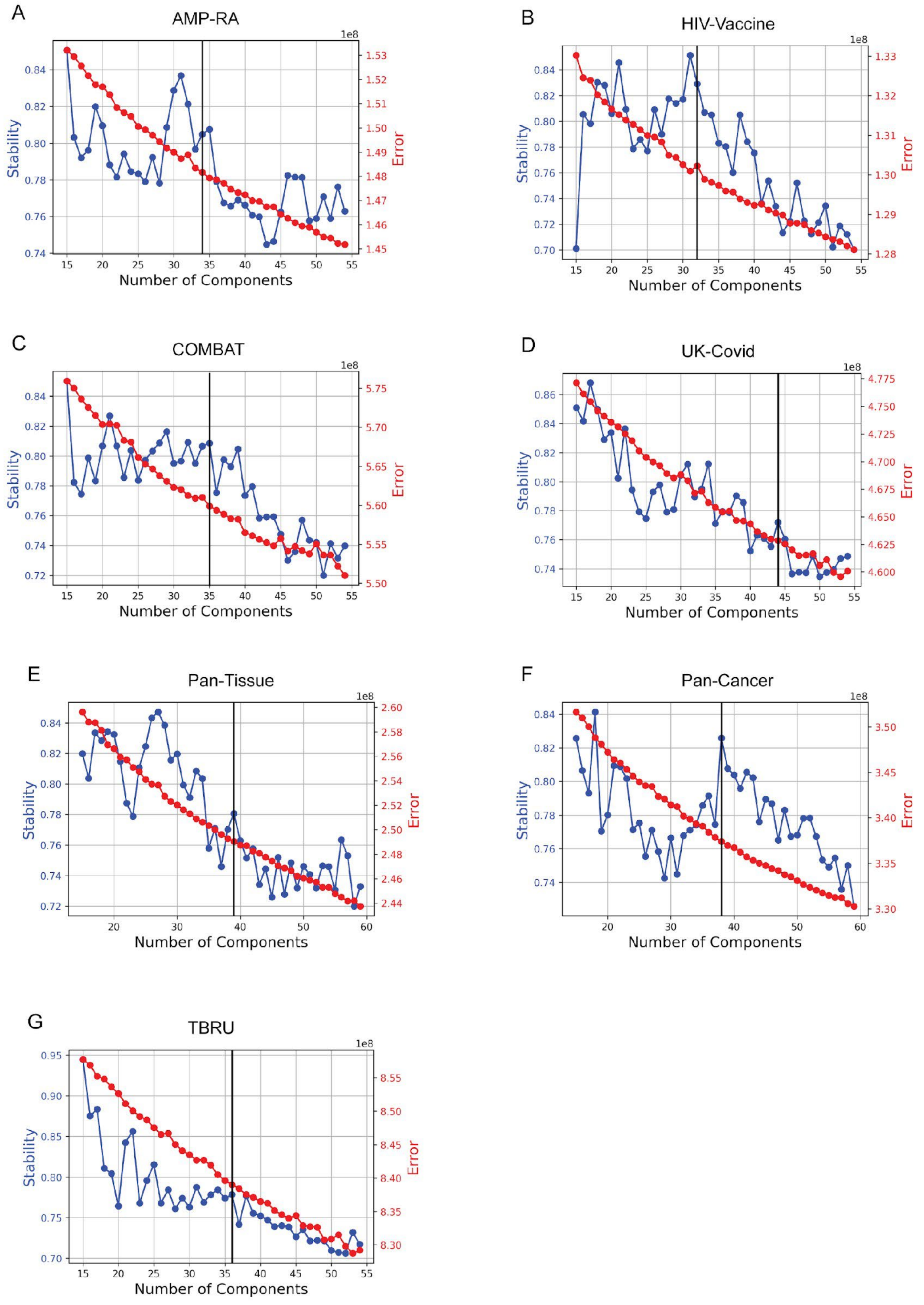

Supplementary Figure 2. K selection plots for consensus non-negative matrix factorization (cNMF) runs on reference datasets. Vertical line denotes the selected number of

components. Frobenius error is shown in red and average Sillhouette score across consensus clusters is shown in blue.

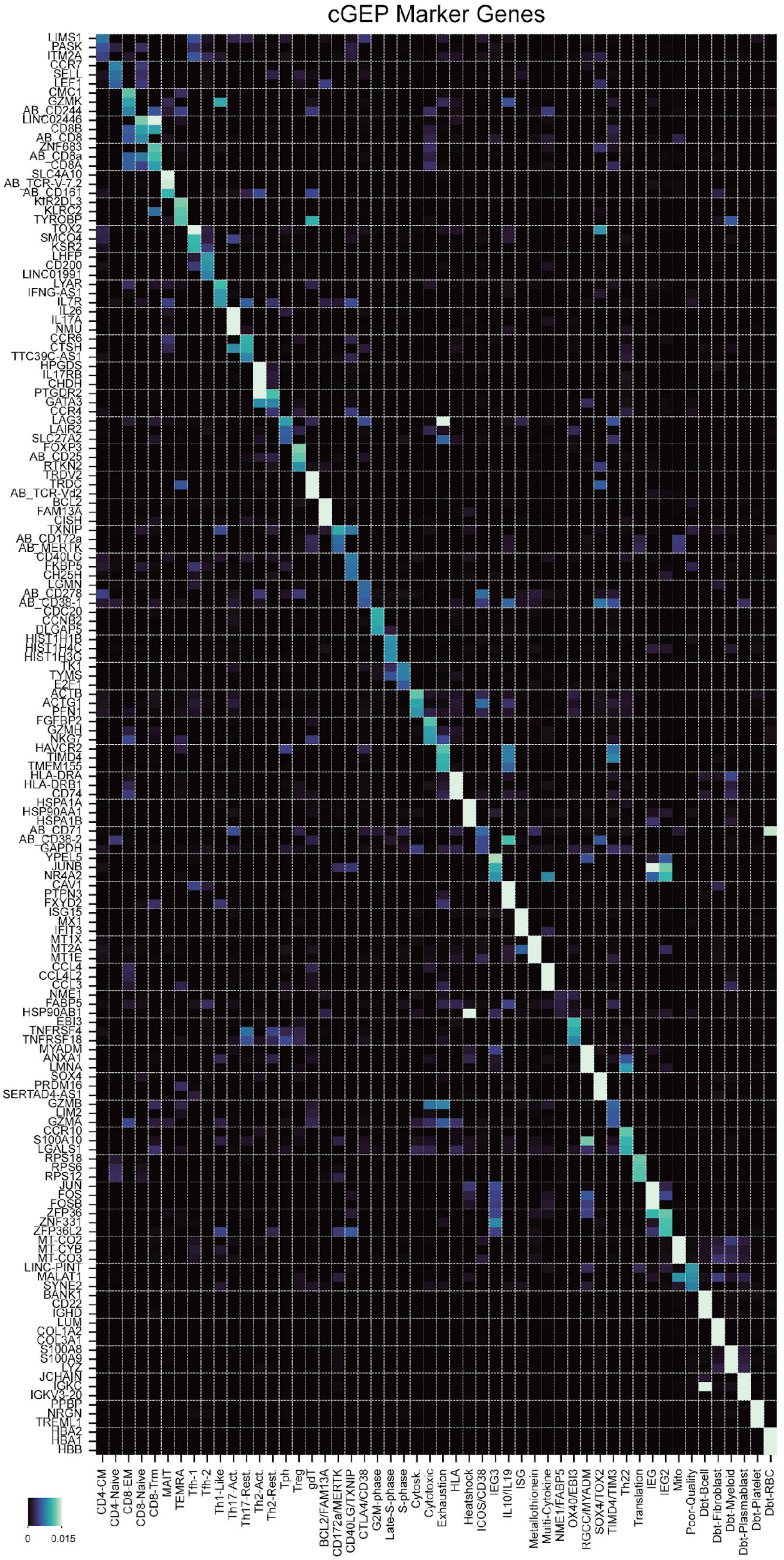
cGEP Marker Genes
LIMS1
PASK
ITM2A
CCR7
SELL
LEF1
CMC1
GZMK
AB_CD244
LINC02446
CD8B
AB_CD8
ZNF683
AB_CD8a
CD8A
SLC4A10
AB_TCR-V-7.2
AB_CD161
KIR2DL3
KLRC2
TYROBP
TOX2
SMCO4
KSR2
LHFP
CD200
LINC01991
LYAR
IFNG-AS1
IL7R
IL26
IL17A
NMU
CCR6
CTSH
TTC39C-AS1
HPGDS
IL17RB
CHDH
PTGDR2
GATA3
CCR4
LAG3
LAIR2
SLC27A2
FOXP3
AB_CD25
RTKN2
TRDV2
TRDC
AB_TCR-Vd2
BCL2
FAM13A
CISH
TXNIP
AB_CD172a
AB_MERTK
CD40LG
FKBP5
CH25H
LGMN
AB_CD278
AB_CD38-1
CDC20
CCNB2
DLGAP5
HIST1H1B
HIST1H4C
HIST1H3G
TK1
TYMS
E2F1
ACTB
ACTG1
PFN1
FGFBP2
GZMH
NKG7
HAVCR2
TIMD4
TMEM155
HLA-DRA
HLA-DRB1
CD74
HSPA1A
HSP90AA1
HSPA1B
AB_CD71
AB_CD38-2
GAPDH
YPEL5
JUNB
NR4A2
CAV1
PTPN3
FXYD2
ISG15
MX1
IFIT3
MT1X
MT2A
MT1E
CCL4
CCL4L2
CCL3
NME1
FABP5
HSP90AB1
EBI3
TNFRSF4
TNFRSF18
MYADM
ANXA1
LMNA
SOX4
PRDM16
SERTAD4-AS1
GZMB
LIM2
GZMA
CCR10
S100A10
LGALS1
RPS18
RPS6
RPS12
JUN
FOS
FOSB
ZFP36
ZNF331
ZFP36L2
MT-CO2
MT-CYB
MT-CO3
LINC-PINT
MALAT1
SYNE2
BANK1
CD22
IGHD
LUM
COL1A2
COL3A1
S100A8
S100A9
LYZ
JCHAIN
IGKC
IGKV3-20
PPBP
NRGN
TREML1
HBA2
HBA1
HBB
CD4-CM
CD4-Naive
CD8-EM
CD8-Naive
CD8-Trm
MAIT
TEMRA
Tfh-1
Tfh-2
Th1-Like
Th17-Act.
Th17-Rest.
Th2-Act.
Th2-Rest.
Tph
Treg
gdT
BCL2/FAM13A
CD172a/MERTK
CD40LG/TXNIP
CTLA4/CD38
G2M-phase
Late-S-phase
S-phase
Cytosk.
Cytotoxic
Exhaustion
HLA
Heatshock
ICOS/CD38
IEG3
IL10/IL19
ISG
Metallothionein
Multi-Cytokine
NME1/FABP5
OX40/EBI3
RGCC/MYADM
SOX4/TOX2
TIMD4/TIM3
Th22
Translation
IEG
IEG2
Mito
Poor-Quality
Dbt-Bcell
Dbt-Fibroblast
Dbt-Myeloid
Dbt-Plasmablast
Dbt-Platelet
Dbt-RBC
0
0.015

Supplementary Figure 3. Example marker genes for all cGEPs. Color indicates average cNMF gene score, which denotes how much 1 additional count of unnormalized usage would be expected to increase Z-scored gene expression.

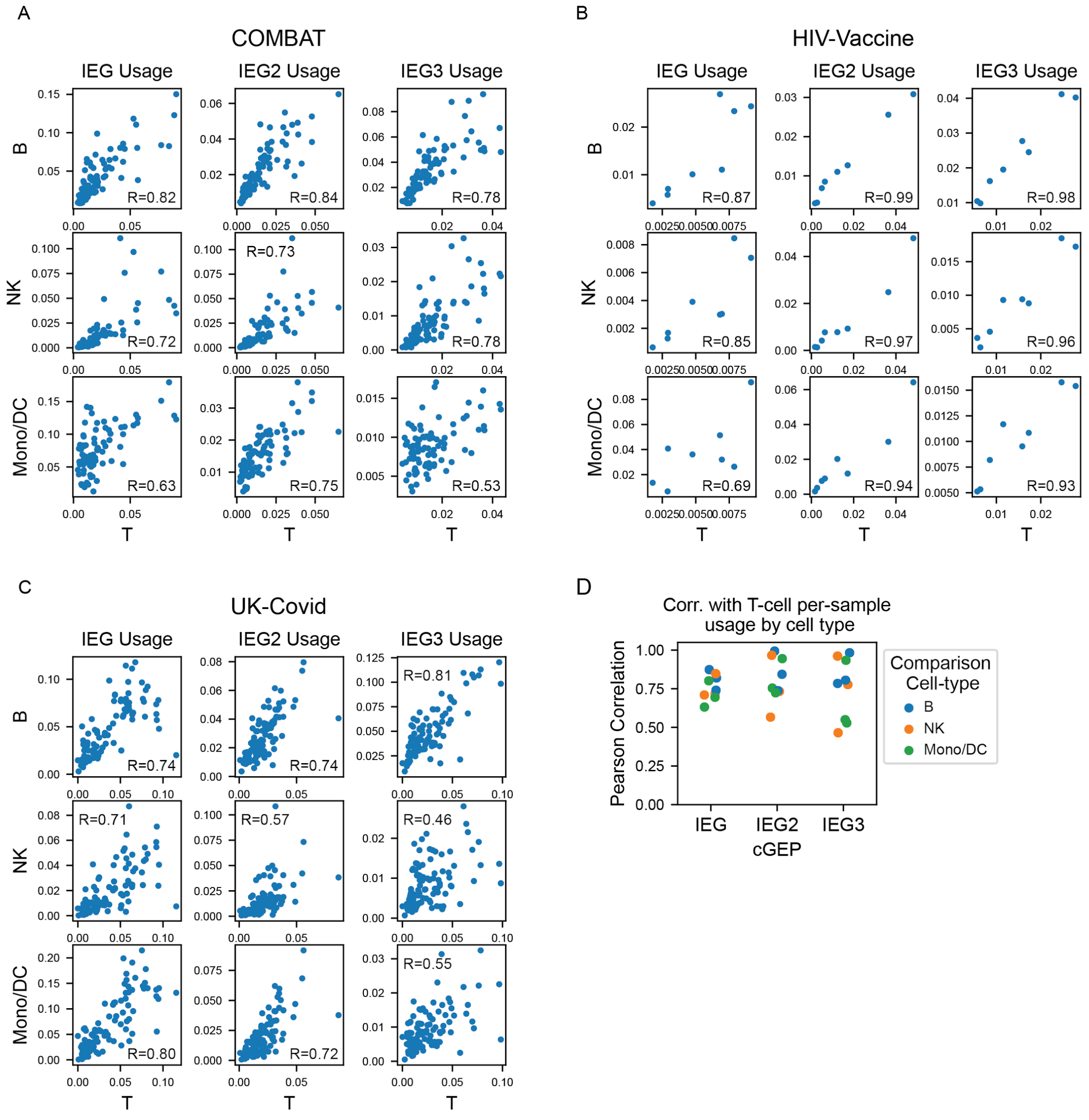

Supplementary Figure 4. Immediate early gene usage across circulating blood cell types.

a-c, Average per-sample usage of each IEG cGEP in T cells versus monocytes and dendritic cells, NK cells, or B-cells, in the three reference PBMC datasets. **d,** Correlation of per-sample average cGEP usage in T cells with that in B-cells, NK-cells for the 3 immediate early gene cGEPs, in the same datasets.

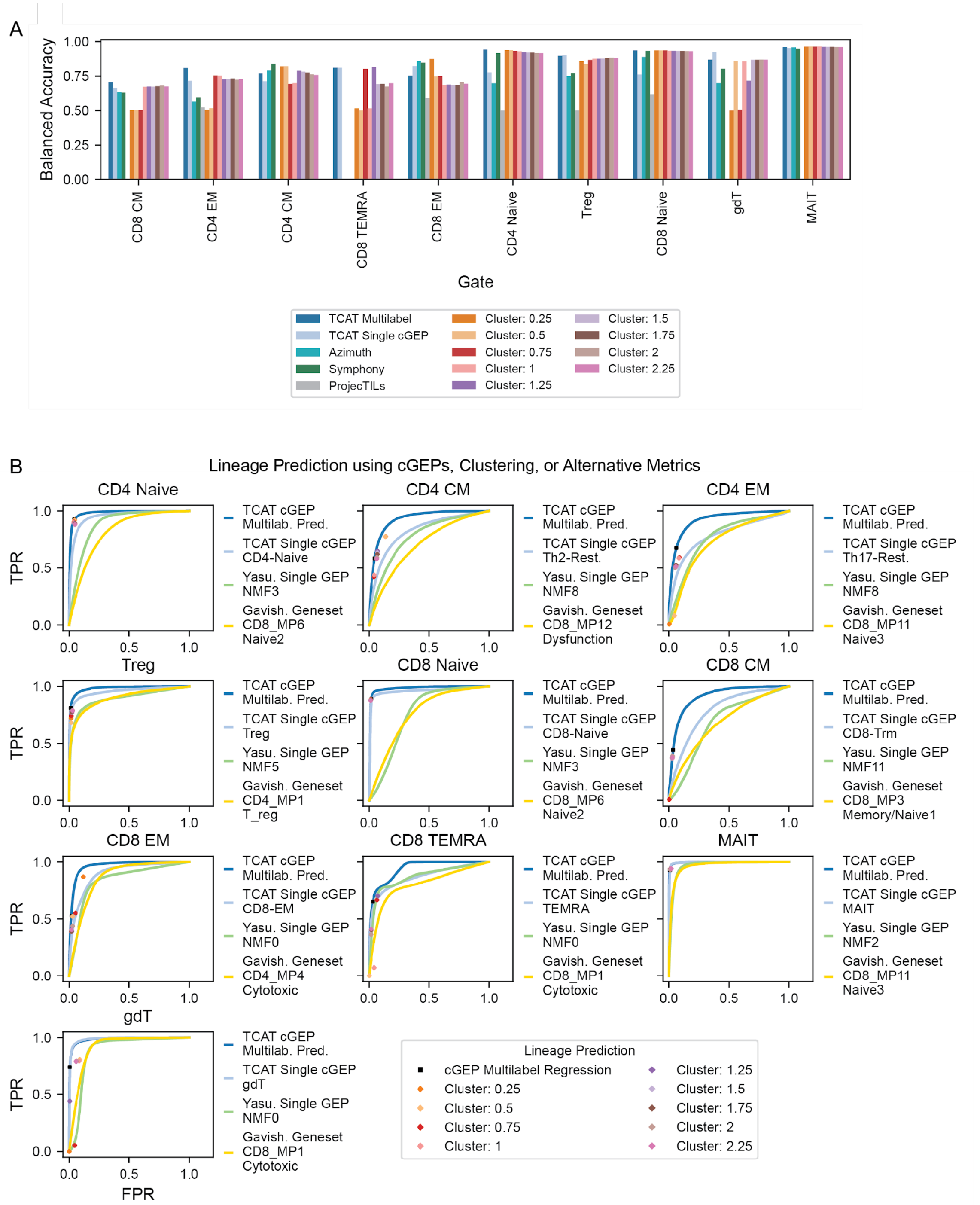

Supplementary Figure 5. Additional TCAT benchmark analyses. a, Comparison of TCAT, Azimuth, Symphony, ProjecTILs, and clustering with multiple Leiden resolution parameters for predicting manually gated subsets in the Flu-Vaccine dataset. TCAT Single cGEP assigned each cell to the subset that was most associated with its most highly used subset cGEP. Clustering assigned each cell to the subset most associated with its cluster (**Methods**). **b,** Receiver

operator curves (ROCs) for prediction of manually gated subset based on TCAT multilabel prediction (dark blue), a single most associated TCAT cGEP (light blue), analogous predictions using the single most associated NMF component published in Yasumizu et al., 2024[16], or using gene sets from NMF components in Gavish et al., 2023[17]. Individual points show accuracies of discrete predictions based on cGEP multilabel regression, or clustering with the Leiden resolution specified in the legend.

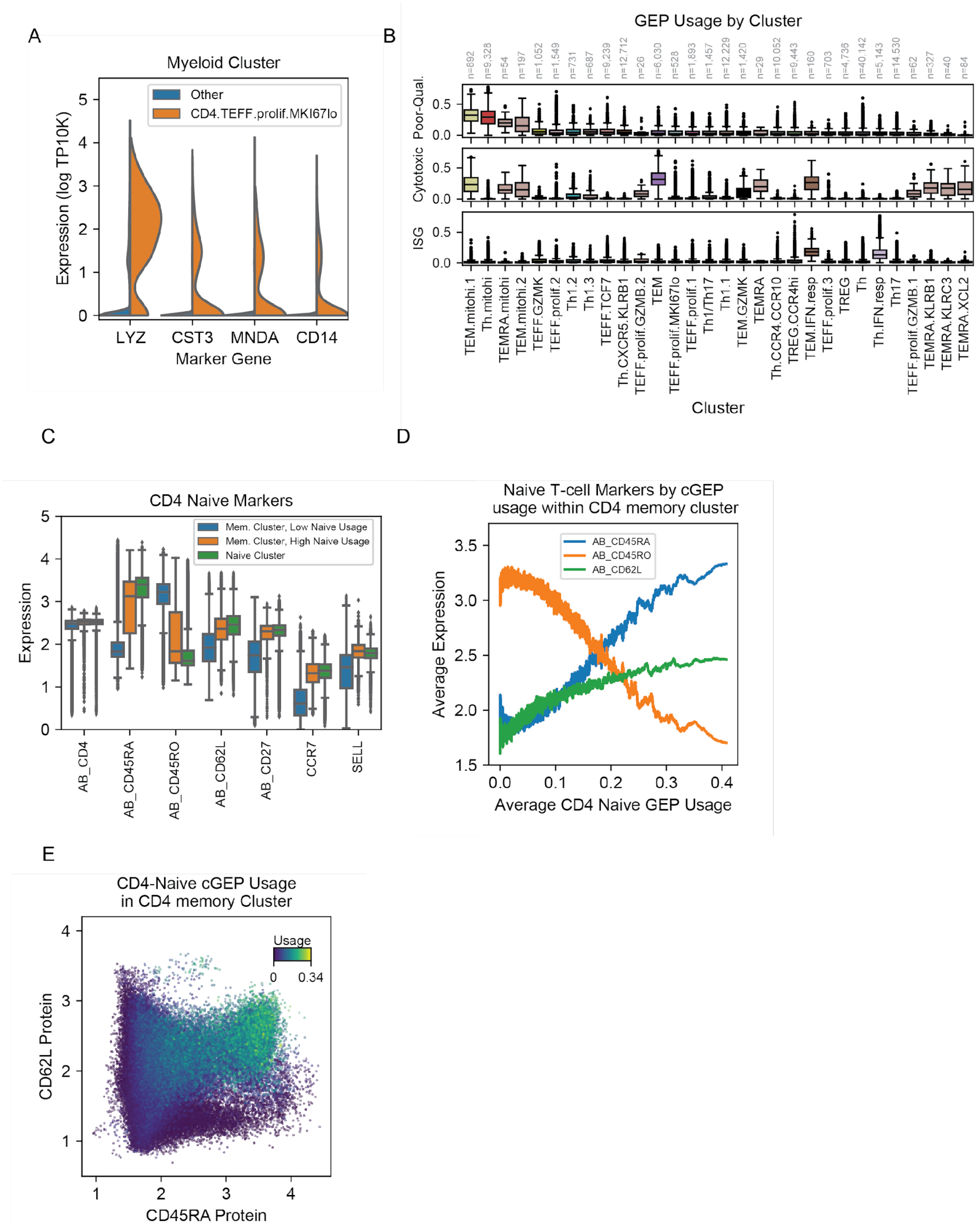

Supplementary Figure 6. Characterization of COMBAT dataset clustering. a, Violin plot for myeloid cell marker genes in cells originally annotated as CD4 memory T cells broken out by the CD4.TEFF.prolif.MKI67lo subcluster, or all other subclusters combined. **b,** Usage of the ISG,

Cytotoxic, and Poor-quality cGEPs in cells stratified by their CD4 memory subcluster. “CD4” is omitted from cluster names for space. Boxes represent the interquartile range and whiskers represent 1.5 x interquartile range. The box center line indicates the median. **c,** Expression of CD4 naive marker genes in cells initially clustered as CD4 memory (blue and orange boxes) or CD4 naive (green cluster). Cells initially clustered as CD4 memory are stratified by their usage of the CD4 naive cGEP with a threshold of 0.1. Boxes represent the interquartile range and whiskers represent 1.5 x interquartile range. The box center line indicates the median. Sample sizes are Mem. Cluster, Low Naive: 140,196 cells; Mem. Cluster, High Naive Usage: 5,279 cells; Naive Cluster: 123,291 cells. **d,** Average expression of naive and memory T cell markers in cells clustered as CD4 memory in a sliding window of 200 cells, ordered by usage of the CD4-Naive cGEP. **e,** Expression of CD45RA and CD62L colored by usage of the CD4-Naive cGEP.

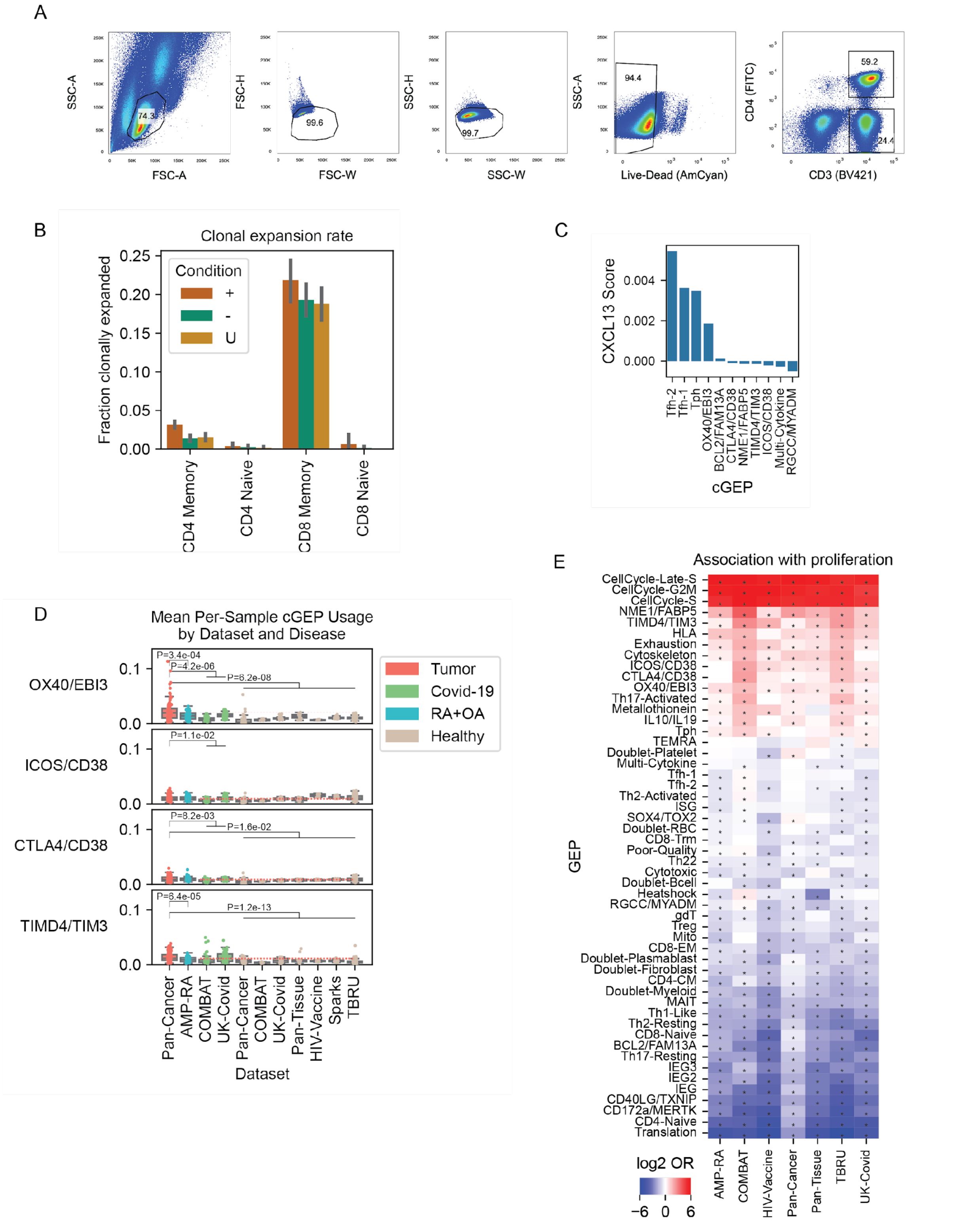

Supplementary Figure 7. cGEP associations with proliferation across datasets. a, Gating strategy to identify CD3+ CD4+ and CD3+ CD4- populations in the AIM-Seq experiment. **b,** Fraction of clonally expanded T cells stratified by memory vs. naive and by treatment condition.

Clonal expansion is defined as having 2+ T cells in the same donor with the same CDR3 beta chain. Bar value reflects the total observed fraction of expanded clones, while error bars reflect 95 percent bootstrap confidence intervals (1000 bootstraps) around this measurement. N=12,118 CD4 Memory clones, N=8197 CD4 Naive clones, N=2945 CD8 Memory clones, N=4447 CD8 Naive clones. **c,** Gene weights for *CXCL13* in various cGEPs, including Tfh-1, Tfh-2, Tph (positive controls) and all AIM-associated cGEPs. **d,** Boxplots of mean per-sample usage of the AIM-associated cGEPs used to define ASA, by dataset and by disease. Two-sided T-tests were performed for each cGEP to compare differences between tumor samples and other disease types. P-values are Bonferroni adjusted. Boxes represent the interquartile range and whiskers represent 1.5 x interquartile range. The box center line indicates the median. Number of samples shown include n=89 Tumor/Pan-Cancer; n=81 RA+OA; n=77, n=80, Covid-19 from COMBAT, UK-Covid, respectively; n=47, n=10, n=21, n=12, n=8, n=41, n=259 Healthy from Pan-Cancer, COMBAT, UK-Covid, Pan-Tissue, HIV-Vaccine, Sparks, TBRU, respectively. **e,** Heatmap of the average Log2 ratio of mean usage in proliferating cells (usage>0.1 of proliferation GEPs) and non-proliferating cells (usage<0.1) for all GEPs (rows) and datasets (columns). An absolute value ceiling of 6 is used to aid visualization. * indicates paired two-sided T-test P<.05.

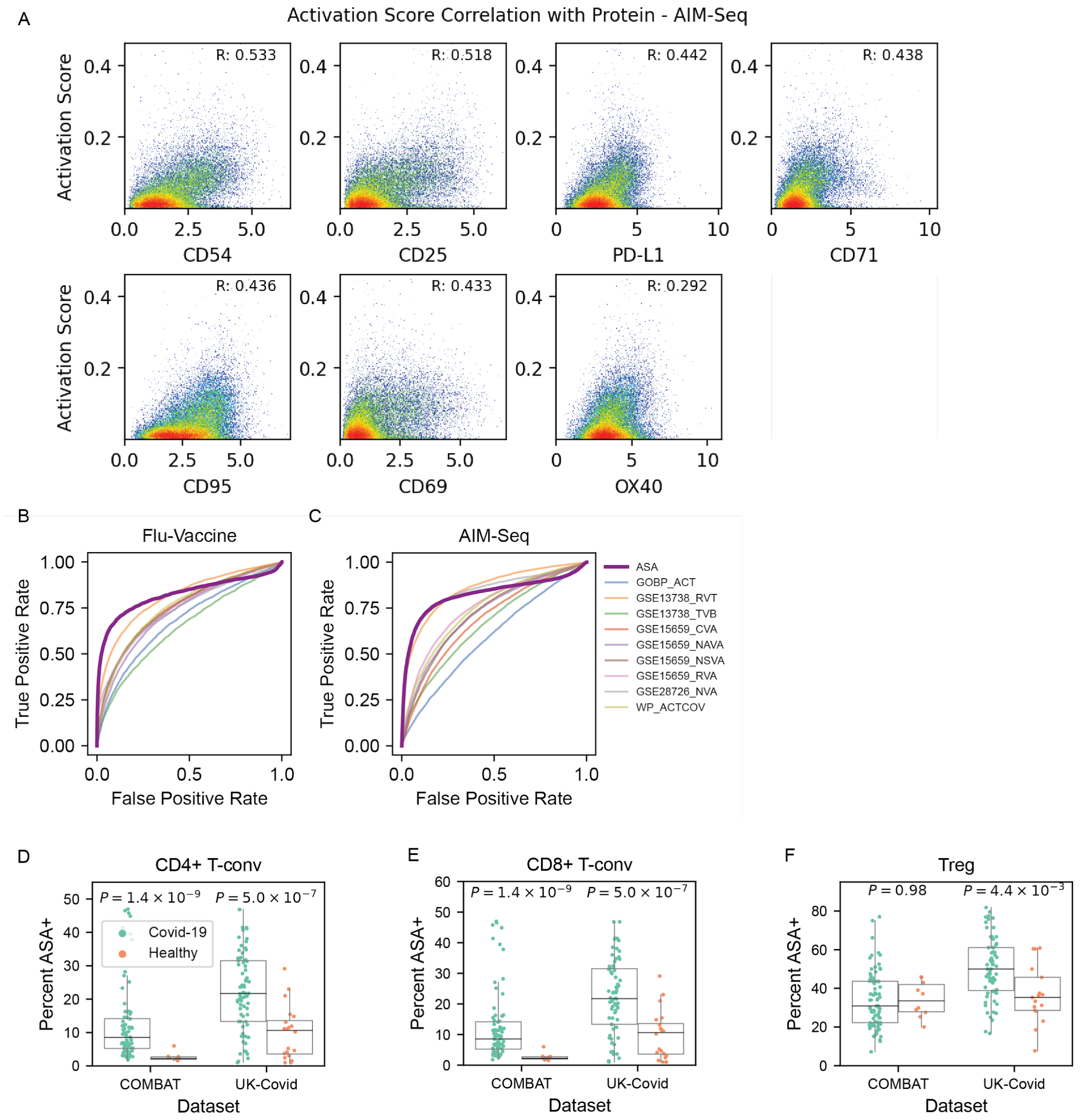

Supplementary Figure 8. Additional antigen-specific activation analyses. a, Antigen-specific activation (ASA) score correlation with surface protein activation markers in the AIM-Seq dataset. **b,** ROC curves for ASA prediction of CD71+CD95+ in the Flu-Vaccine dataset, as compared to prediction using per-cell gene scoring with T cell activation gene sets. **c,** Same as **b** for AIM-positivity prediction in the AIM-Seq dataset. Full gene set names (in order) are GOBP_ACTIVATED_T_CELL_PROLIFERATION, GSE13738_RESTING_VS_TCR_ACTIVATED_CD4_TCELL_DN,

GSE13738_TCR_VS_BYSTANDER_ACTIVATED_CD4_TCELL_UP,
GSE15659_CD45RA_NEG_CD4_TCELL_VS_ACTIVATED_TREG_DN,
GSE15659_NAIVE_CD4_TCELL_VS_ACTIVATED_TREG_DN,
GSE15659_NONSUPPRESSIVE_TCELL_VS_ACTIVATED_TREG_DN,
GSE15659_RESTING_VS_ACTIVATED_TREG_DN,
GSE28726_NAIVE_VS_ACTIVATED_CD4_TCELL_DN,
WP_TCELL_ACTIVATION_SARSCOV2. **d-f,** Percentage of activated CD4 Convs, CD8 Convs, and Tregs based on ASA>0.065 in Covid-19 and healthy control samples from COMBAT and UK-Covid datasets. Boxes represent the interquartile range and whiskers represent 95% quantile range. P-values are from a T-test of pseudobulk per-sample average usage between Covid-19 and healthy samples (COMBAT: n=77 Covid-19, 10 Healthy, UK-Covid: n=80 Covid-19, 21 Healthy).

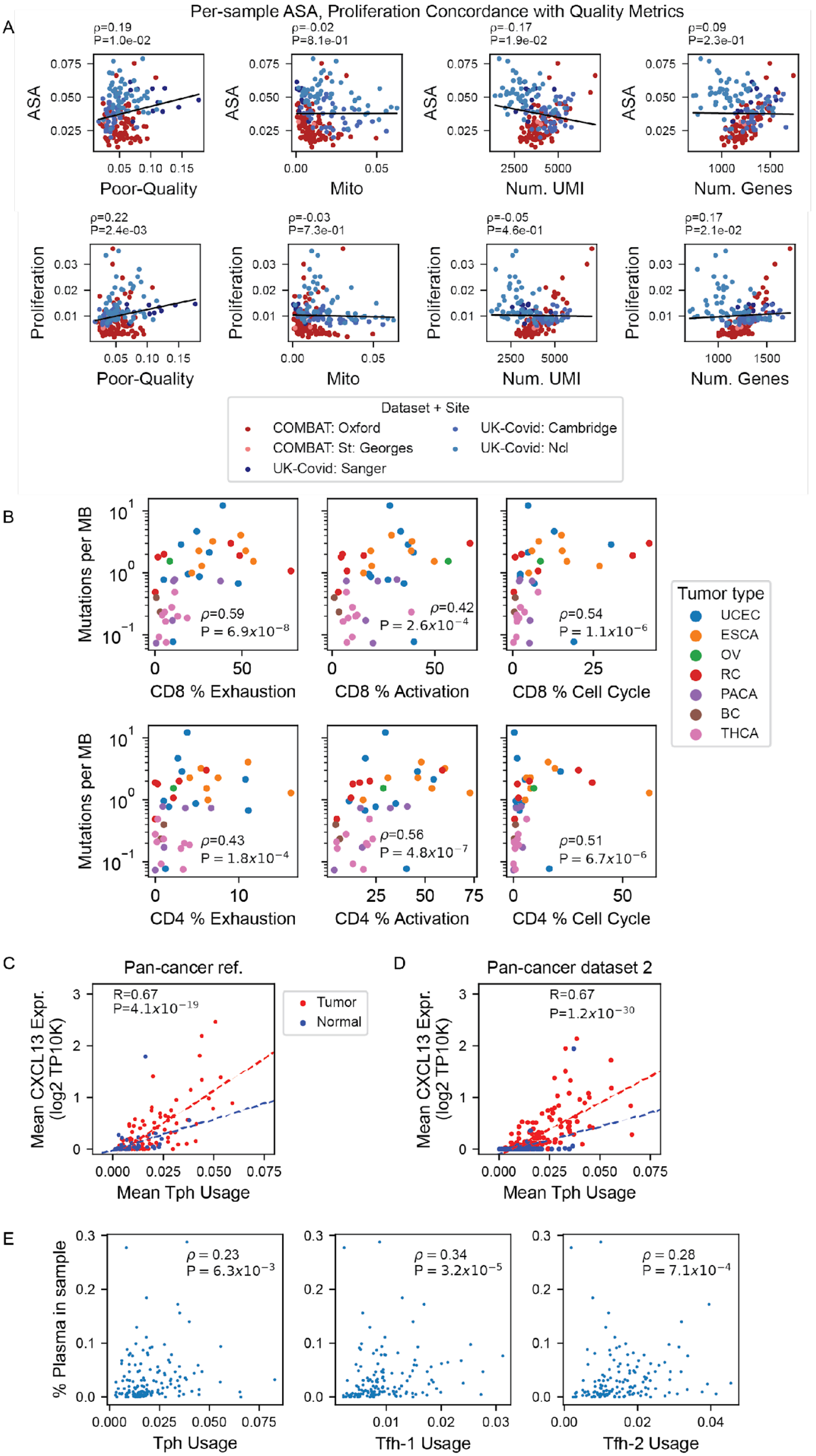

Per-sample ASA, Proliferation Concordance with Quality Metrics
A
ρ=0.19 P=1.0e-02
ρ=-0.02 P=8.1e-01
ρ=-0.17 P=1.9e-02
ρ=0.09 P=2.3e-01
ASA
Poor-Quality
Mito
Num. UMI
Num. Genes
ρ=0.22 P=2.4e-03
ρ=-0.03 P=7.3e-01
ρ=-0.05 P=4.6e-01
ρ=0.17 P=2.1e-02
Proliferation
Dataset + Site
COMBAT: Oxford
COMBAT: St. Georges
UK-Covid: Sanger
UK-Covid: Cambridge
UK-Covid: Ncl
B
Mutations per MB
ρ=0.59 P = 6.9x10^-8
ρ=0.42 P = 2.6x10^-4
ρ=0.54 P = 1.1x10^-6
CD8 % Exhaustion
CD8 % Activation
CD8 % Cell Cycle
Tumor type
UCEC
ESCA
OV
RC
PACA
BC
THCA
ρ=0.43 P = 1.8x10^-4
ρ=0.56 P = 4.8x10^-7
ρ=0.51 P = 6.7x10^-6
CD4 % Exhaustion
CD4 % Activation
CD4 % Cell Cycle
C
Pan-cancer ref.
R=0.67 P=4.1x10^-19
Tumor
Normal
Mean CXCL13 Expr. (log2 TP10K)
Mean Tph Usage
D
Pan-cancer dataset 2
R=0.67 P=1.2x10^-30
E
% Plasma in sample
ρ = 0.23 P = 6.3x10^-3
ρ = 0.34 P = 3.2x10^-5
ρ = 0.28 P = 7.1x10^-4
Tph Usage
Tfh-1 Usage
Tfh-2 Usage

Supplementary Figure 9. Additional activation analyses and Tph/Tfh cells in cancer. a, Per-sample scatterplots showing concordance between mean antigen-specific activation (ASA) score (top) or proliferation (bottom) and various quality metrics, across datasets and sites. Spearman correlation and two-sided P-values are shown. **b,** Per-individual scatterplots of concordance between tumor mutation burden and percentage of exhausted (left), activated (middle), and proliferating (right) T cells for both CD8 (top) and CD4 (bottom) T cells. Tumor mutation burden is defined as the average number of mutations per MB. Spearman correlation and two-sided P-values are shown. **c-d,** Average usage of the T peripheral helper (Tph) cGEP compared to average *CXCL13* expression from T cells within tumors and matched normal tissue samples in the primary Pan-cancer reference and Luo et al., 2022[15]. Trend lines show the regression coefficients fit for tumors and normal samples separately. Pearson correlation and two-sided P-values are shown. **e,** Percentage of cells annotated as plasma cells against the average Tph, Tfh-1, or Tfh-2 usage within T cells from tumor samples in Luo et al., 2022[15]. Spearman correlation and two-sided P-values are shown.

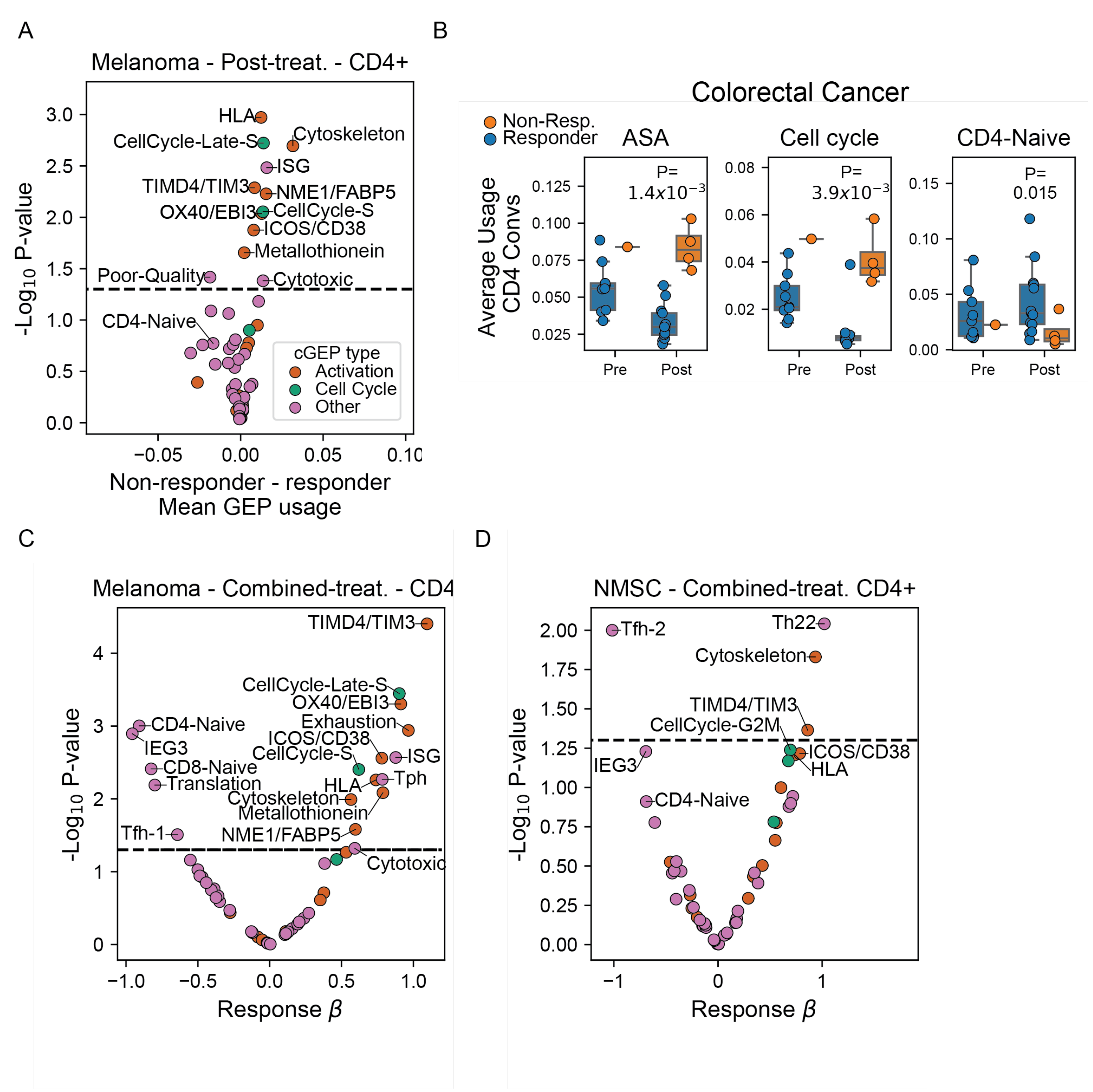

Supplementary Figure 10. Additional immune checkpoint inhibitor analyses. a, Association of cGEP usage with ICI response in CD4 T cells for post-treatment melanoma samples. P-values are from a two-tailed T-test. Dots are colored by cGEP type. **b**, Average ASA and cell cycle score and CD4-Naive cGEP usage in CD4 T cells from CRC stratified by ICI response (color) and Pre- vs. Post- treatment. P-values are from a one-tailed T-test. Boxes represent the interquartile range and whiskers represent 1.5 x interquartile range. The box

center line indicates the median. **c-d,** Associations of cGEP usage between responders versus non-responders, in CD4 T cells of combined pre- and post- treatment tumors controlling for treatment timepoint and donor of origin as fixed and random effects, respectively. Betas were calculated using a mixed linear model associating pseudobulk average cGEP usage with immunotherapy response. P-values were calculated with a likelihood ratio test.

Supplementary Tables

Supplementary Table 1. Dataset Information and cGEP Summary. The first table contains a summary of each reference dataset analyzed. The second table contains a summary of cGEPs including their full name, abbreviated name, assigned class, top 3 most strongly associated genes, and which datasets they were derived from.

Supplementary Table 2. Marker genes. Top 200 marker genes for each cGEP ranked by average Z-score normalized gene spectra score with cells colored by this score.

Supplementary Table 3. Gene-set enrichment. The “GO_Enrichment” tab includes the top 10 associated gene sets for each cGEP including the GEP name, gene-set name, fisher exact test odds ratio, and P-value. The subsequent tabs include the same information but for enrichment tests for gene sets defined from a dataset that polarized T cells for either 16 hours (16h) or 5 days (5d) starting from either naive (TN) or memory T cells (TM).

Supplementary Table 4. Correlation with cell quality features. Each tab includes the Pearson correlation of each cGEP’s usage (rows) with different per-cell quality features for each dataset (columns). MitoFrac denotes the % of unique molecular identifiers from MT-genes. RNA_Detected denotes the number of unique genes detected per cell. RNA_Count denotes the number of unique molecular identifiers per cell. PCFrac denotes the percentage of unique molecular identifiers that are assigned to a protein coding gene in Gencode version 44.

Supplementary Table 5. AIM-Seq association. Regression coefficients and P-values for the association between cGEP usage and binary variables reflecting CEFX vs. mock stimulation or

AIM-positive vs. AIM-negative. Coef. represents the regression coefficient, P represents the P-value, and Q represents the FDR-corrected P-value.

Supplementary Table 6. Association with proliferation. T-statistics, P-values, and $\log_2$ odds ratios for the paired T-test of proliferating and non-proliferating T cells in each dataset (tabs). For the meta-analysis across datasets it provides the Fisher's method combined P-value and the average $\log_2$ odds ratio.

Supplementary Table 7. Association with disease. Ordinary least squares regression coefficients (Beta), P-values (P), FDR-corrected Q-values (Q), and average fold changes (FC) for phenotype associations shown in Figure S7.

Supplementary Table 8. Association with immune checkpoint inhibitor response. P-values and effect sizes for associations with ICI in melanoma, non-melanoma skin cancer (NMSC), and colorectal cancer (CRC). Results for pre-treatment (Pre) or post-treatment (Post) samples only are computed as two-tailed T-tests. Results for the combination of Pre and Post samples (Both) is computed from linear regression controlling for timepoint of the sample as a covariate. Meta-analysis P-values are computed using Fisher's Method.

Supplementary Note References

1. Gene Ontology Consortium *et al.* The Gene Ontology knowledgebase in 2023. *Genetics*

224, (2023).

2. Cano-Gamez, E. *et al.* Single-cell transcriptomics identifies an effectorness gradient shaping the response of CD4+ T cells to cytokines. *Nat. Commun.* **11**, 1801 (2020).
3. Arner, E. *et al.* Transcribed enhancers lead waves of coordinated transcription in transitioning mammalian cells. *Science* **347**, 1010–1014 (2015).
4. Vacca, A. *et al.* Conserved temporal ordering of promoter activation implicates common mechanisms governing the immediate early response across cell types and stimuli. *Open Biol.* **8**, (2018).
5. Bahrami, S. & Drabløs, F. Gene regulation in the immediate-early response process. *Adv. Biol. Regul.* **62**, 37–49 (2016).
6. van den Brink, S. C. *et al.* Single-cell sequencing reveals dissociation-induced gene expression in tissue subpopulations. *Nat. Methods* **14**, 935–936 (2017).
7. Lacar, B. *et al.* Nuclear RNA-seq of single neurons reveals molecular signatures of activation. *Nat. Commun.* **7**, 11022 (2016).
8. Stephenson, E. *et al.* Single-cell multi-omics analysis of the immune response in COVID-19. *Nat. Med.* **27**, 904–916 (2021).
9. COvid-19 Multi-omics Blood ATlas (COMBAT) Consortium. Electronic address: julian.knight@well.ox.ac.uk & COvid-19 Multi-omics Blood ATlas (COMBAT) Consortium. A blood atlas of COVID-19 defines hallmarks of disease severity and specificity. *Cell* **185**, 916–938.e58 (2022).
10. Asashima, H. *et al.* PD-1highCXCR5-CD4+ peripheral helper T cells promote CXCR3+ plasmablasts in human acute viral infection. *Cell Rep.* **42**, 111895 (2023).
11. Zhang, F. *et al.* Deconstruction of rheumatoid arthritis synovium defines inflammatory subtypes. *Nature* **623**, 616–624 (2023).

12. Sun, J. *et al.* Metallothionein-1 suppresses rheumatoid arthritis pathogenesis by shifting the

Th17/Treg balance. *Eur. J. Immunol.* **48**, 1550–1562 (2018).

13. Rao, D. A. *et al.* Pathologically expanded peripheral T helper cell subset drives B cells in rheumatoid arthritis. *Nature* **542**, 110–114 (2017).

14. Miyazaki, Y. *et al.* Th22 Cells Promote Osteoclast Differentiation via Production of IL-22 in Rheumatoid Arthritis. *Front. Immunol.* **9**, 2901 (2018).

15. Luo, H. *et al.* Pan-cancer single-cell analysis reveals the heterogeneity and plasticity of cancer-associated fibroblasts in the tumor microenvironment. *Nat. Commun.* **13**, 6619 (2022).

16. Yasumizu, Y. *et al.* Single-cell transcriptome landscape of circulating CD4+ T cell populations in autoimmune diseases. *Cell Genomics* 100473 (2024) doi:10.1016/j.xgen.2023.100473.

17. Gavish, A. *et al.* Hallmarks of transcriptional intratumour heterogeneity across a thousand tumours. *Nature* **618**, 598–606 (2023).
