# Supplementary material for: Reproducible single-cell annotation of programs underlying T cell subsets, activation states and functions
Source: Nat Methods. 2025 Sep 3;22(9):1964–80. doi: 10.1038/s41592-025-02793-1 (PMC12446064; doi:10.1038/s41592-025-02793-1)
Supplement: Supplementary file 2 — Reporting Summary [file 41592_2025_2793_MOESM2_ESM.pdf]

nature portfolio

Corresponding author(s): Soumya Raychaudhuri

Last updated by author(s): Apr 29, 2025

Reporting Summary

Nature Portfolio wishes to improve the reproducibility of the work that we publish. This form provides structure for consistency and transparency in reporting. For further information on Nature Portfolio policies, see our Editorial Policies and the Editorial Policy Checklist.

Statistics

For all statistical analyses, confirm that the following items are present in the figure legend, table legend, main text, or Methods section.

n/a	Confirmed
☐	☒ The exact sample size (*n*) for each experimental group/condition, given as a discrete number and unit of measurement
☐	☒ A statement on whether measurements were taken from distinct samples or whether the same sample was measured repeatedly
☐	☒ The statistical test(s) used AND whether they are one- or two-sided<br>*Only common tests should be described solely by name; describe more complex techniques in the Methods section.*
☐	☒ A description of all covariates tested
☐	☒ A description of any assumptions or corrections, such as tests of normality and adjustment for multiple comparisons
☐	☒ A full description of the statistical parameters including central tendency (e.g. means) or other basic estimates (e.g. regression coefficient) AND variation (e.g. standard deviation) or associated estimates of uncertainty (e.g. confidence intervals)
☐	☒ For null hypothesis testing, the test statistic (e.g. *F*, *t*, *r*) with confidence intervals, effect sizes, degrees of freedom and *P* value noted<br>*Give P values as exact values whenever suitable.*
☐	☒ For Bayesian analysis, information on the choice of priors and Markov chain Monte Carlo settings
☐	☒ For hierarchical and complex designs, identification of the appropriate level for tests and full reporting of outcomes
☐	☒ Estimates of effect sizes (e.g. Cohen's *d*, Pearson's *r*), indicating how they were calculated

Our web collection on statistics for biologists contains articles on many of the points above.

Software and code

Policy information about availability of computer code

Data collection

BD FACSDiva 9.4 was used for FACS sorting cells prior to sequencing.

Data analysis

The code for star-CellAnnotator (starCAT) is available at https://github.com/immunogenomics/starCAT. The analysis scripts used in this paper are available at https://github.com/immunogenomics/TCAT_analysis.

Softwares used in these analysis scripts include:
cNMF 1.5.0
scsim2
anndata 0.10.9
anyio 4.3.0
argon2-cffi 23.1.0
argon2-cffi-bindings 21.2.0
array_api_compat 1.8
arrow 1.3.0
asttokens 2.4.1
async-lru 2.0.4
attrs 23.2.0
Babel 2.14.0
backports.tarfile 1.2.0
beautifulsoup4 4.12.3
bleach 6.1.0

blinker	1.8.2
Brotli	1.1.0
cached-property	1.5.2
certifi	2024.8.30
cffi	1.16.0
charset-normalizer	3.3.2
click	8.1.7
cloudpickle	3.0.0
cnmf	1.5.4
colorama	0.4.6
colorcet	3.1.0
comm	0.2.2
contourpy	1.2.1
cryptography	43.0.0
cycler	0.12.1
dask	2024.5.0
datashader	0.16.1
debugpy	1.8.1
decorator	5.1.1
defusedxml	0.7.1
Deprecated	1.2.14
docutils	0.21.2
entrypoints	0.4
et-xmlfile	1.1.0
exceptiongroup	1.2.2
executing	2.0.1
fastcluster	1.2.6
fastjsonschema	2.19.1
Flask	3.0.3
fonttools	4.53.1
fqdn	1.5.1
fsspec	2024.3.1
future	1.0.0
get-annotations	0.1.2
graphtools	1.5.3
gseapy	1.1.3
h11	0.14.0
h2	4.1.0
h5py	3.11.0
harmonypy	0.0.9
hpack	4.0.0
httpcore	1.0.5
httpx	0.27.0
hyperframe	6.0.1
idna	3.8
igraph	0.11.5
importlib_metadata	7.1.0
importlib_resources	6.4.0
ipykernel	6.29.3
ipython	8.22.2
ipython-genutils	0.2.0
ipywidgets	8.1.2
isoduration	20.11.0
itsdangerous	2.2.0
jaraco.classes	3.4.0
jaraco.context	5.3.0
jaraco.functools	4.0.1
jedi	0.19.1
jeepney	0.8.0
Jinja2	3.1.4
joblib	1.4.2
json5	0.9.25
jsonpointer	2.4
jsonschema	4.22.0
jsonschema-specifications	2023.12.1
jupyter	1.0.0
jupyter_client	7.4.9
jupyter-console	6.6.3
jupyter_core	5.7.2
jupyter-events	0.10.0
jupyter-lsp	2.2.5
jupyter_server	2.14.0
jupyter_server_terminals	0.5.3
jupyterlab	4.2.0
jupyterlab_pygments	0.3.0
jupyterlab_server	2.27.1

jupyterlab_widgets	3.0.10
keyring	25.2.1
kiwisolver	1.4.5
legacy-api-wrap	1.4
leidenalg	0.10.2
llvmlite	0.43.0
locket	1.0.0
magic-impute	3.0.0
markdown-it-py	3.0.0
MarkupSafe	2.1.5
matplotlib	3.8.4
matplotlib-inline	0.1.7
mdurl	0.1.2
mistune	3.0.2
more-itertools	10.3.0
mudata	0.2.3
multipledispatch	0.6.0
munkres	1.1.4
muon	0.1.6
natsort	8.4.0
nbclassic	1.0.0
nbclient	0.10.0
nbconvert	7.16.4
nbformat	5.10.4
nest_asyncio	1.6.0
networkx	3.3
nh3	0.2.18
notebook	6.5.6
notebook_shim	0.2.4
numba	0.60.0
numpy	1.26.4
openpyxl	3.1.2
overrides	7.7.0
packaging	24.1
palettable	3.3.3
pandas	2.2.2
pandocfilters	1.5.0
param	2.1.0
parso	0.8.4
partd	1.4.2
patsy	0.5.6
pexpect	4.9.0
pickleshare	0.7.5
pillow	10.4.0
pip	24.0
pkginfo	1.10.0
pkgutil_resolve_name	1.3.10
platformdirs	4.2.1
ply	3.11
prometheus_client	0.20.0
prompt-toolkit	3.0.42
protobuf	4.25.3
psutil	5.9.8
ptyprocess	0.7.0
pure-eval	0.2.2
pycparser	2.22
pyct	0.5.0
Pygments	2.18.0
PyGSP	0.5.1
pynndescent	0.5.12
pyparsing	3.1.2
PyQt5	5.15.9
PyQt5-sip	12.12.2
PySocks	1.7.1
python-dateutil	2.9.0.post0
python-json-logger	2.0.7
pytz	2024.1
PyYAML	6.0.2
pyzmq	24.0.1
qtconsole	5.5.2
QtPy	2.4.1
readme_renderer	44.0
referencing	0.35.1
requests	2.32.3
requests-toolbelt	1.0.0
rfc3339-validator	0.1.4

rfc3986	2.0.0
rfc3986-validator	0.1.1
rich	13.7.1
rpds-py	0.18.1
scanpy	1.10.1
scikit-learn	1.5.1
scikit-misc	0.1.4
scipy	1.14.1
scprep	1.2.3
seaborn	0.13.2
SecretStorage	3.3.3
Send2Trash	1.8.3
session_info	1.0.0
setuptools	69.5.1
sip	6.7.12
six	1.16.0
sniffio	1.3.1
soupsieve	2.5
stack-data	0.6.2
starcatpy	1.0.8
statsmodels	0.14.1
stdlib-list	0.10.0
tasklogger	1.2.0
terminado	0.18.1
texttable	1.7.0
threadpoolctl	3.5.0
tinycss2	1.3.0
toml	0.10.2
tomli	2.0.1
toolz	0.12.1
tornado	6.4
tqdm	4.66.5
traitlets	5.14.3
twine	5.1.1
types-python-dateutil	2.9.0.20240316
typing_extensions	4.11.0
typing-utils	0.1.0
tzdata	2024.1
umap-learn	0.5.5
unicodedata2	15.1.0
uri-template	1.3.0
urllib3	2.2.2
wcwidth	0.2.13
webcolors	1.13
webencodings	0.5.1
websocket-client	1.8.0
Werkzeug	3.0.3
wheel	0.43.0
widgetsnbextension	4.0.10
wrapt	1.16.0
xarray	2024.3.0
xlrd	1.2.0
zipp	3.17.0

For manuscripts utilizing custom algorithms or software that are central to the research but not yet described in published literature, software must be made available to editors and reviewers. We strongly encourage code deposition in a community repository (e.g. GitHub). See the Nature Portfolio guidelines for submitting code & software for further information.

Data

Policy information about availability of data

All manuscripts must include a data availability statement. This statement should provide the following information, where applicable:

- Accession codes, unique identifiers, or web links for publicly available datasets
- A description of any restrictions on data availability
- For clinical datasets or third party data, please ensure that the statement adheres to our policy

The data used in this study for training and validating TCAT is publicly available, and can be downloaded from the following sources: https://doi.org/10.7303/syn52297840 (AMP-RA), https://zenodo.org/records/5461803 (Pan-Cancer), GEO: GSE164378 (HIV-Vaccine), https://www.ebi.ac.uk/biostudies/arrayexpress/studies/E-MTAB-10026 (UK-Covid), https://zenodo.org/records/6120249 (COMBAT), https://www.tissueimmunecellatlas.org/ (Pan-Tissue), GEO: GSE158769 (TBRU), GEO: GSE206265 (Flu-Vaccine). The count matrices for the Activation Induced Marker (AIM)-Seq data produced in this study are located on Zenodo (https://zenodo.org/records/15271929).

Human research participants

Policy information about studies involving human research participants and Sex and Gender in Research.

Reporting on sex and gender	All five participants were female. No sex-specific analyses were performed.
Population characteristics	Participants were healthy adults between the ages 40 and 50. We excluded individuals with autoimmune diseases or on immunomodulatory medications.
Recruitment	These individuals were recruited from the Partners Biobank. We excluded individuals with autoimmune diseases or on immunomodulatory medications. Recruitment occurred at clinics associated with MGB and may be biased towards more complex cases and individuals representative of the Greater Boston area. Self-selection biases may be present as partaking in Partners Biobank is optional.
Ethics oversight	Mass General Brigham Institutional Review Board (IRB)

Note that full information on the approval of the study protocol must also be provided in the manuscript.

Field-specific reporting

Please select the one below that is the best fit for your research. If you are not sure, read the appropriate sections before making your selection.

[x] Life sciences [ ] Behavioural & social sciences [ ] Ecological, evolutionary & environmental sciences

For a reference copy of the document with all sections, see nature.com/documents/nr-reporting-summary-flat.pdf

Life sciences study design

All studies must disclose on these points even when the disclosure is negative.

Sample size	Our primary analysis of public data included 1.7 million T cells from 905 samples from 695 individuals. This allowed us to define a comprehensive atlas of T cell states in diseases using some of the largest scRNA-seq datasets publicly available for T cells. As we were highly powered to resolve cell states in this large dataset, we then applied it to annotate cell states in our smaller experimental study, which included 43,222 cells across five samples from three stimulation conditions (stimulated, unstimulated, mock). We did not perform an analysis of number of samples necessary prior to performing the experiment. However, this sample size provided us many thousands of cells per donor, per stimulation condition. We were interested in testing the effects of simulation condition on cell states. This number of cells and samples allowed us to be powered to detect significant cell state differences between stimulation conditions.
Data exclusions	No data was excluded from analyses.
Replication	Data was collected on five participants (five replicates). All replications were successful and included in the data analysis. No further replication of data was performed.
Randomization	All cells from all samples were randomly sorted into two groups corresponding to peptide treated and mock-stimulated cells.
Blinding	Blinding was not relevant to the study as all samples were assigned to both peptide treated and mock-stimulated conditions. Data analysis was unbiased and tested differences between antigen-stimulation statuses.

Reporting for specific materials, systems and methods

We require information from authors about some types of materials, experimental systems and methods used in many studies. Here, indicate whether each material, system or method listed is relevant to your study. If you are not sure if a list item applies to your research, read the appropriate section before selecting a response.

Materials & experimental systems

n/a	Involved in the study	
[ ]	[x]	Antibodies
[x]	[ ]	Eukaryotic cell lines
[x]	[ ]	Palaeontology and archaeology
[x]	[ ]	Animals and other organisms
[x]	[ ]	Clinical data
[x]	[ ]	Dual use research of concern

Methods

n/a	Involved in the study	
[x]	[ ]	ChIP-seq
[ ]	[x]	Flow cytometry
[x]	[ ]	MRI-based neuroimaging

Antibodies

Antibodies used

Co-stimulation:
Anti-CD28 antibody, Biolegend, Catalog #: 302933 RRID: AB_11150591
Anti-CD49d antibody, Biolegend, Catalog #: 304339 RRID: AB_281044

Proteogenomics:
TotalSeq™-C Human Universal Cocktail, V1.0, Biolegend, Catalog #: 399905
Human TOTAL-SeqC Repertoire (5') Hashing Antibodies, BioLegend, Catalog #: 394661, 394663, 394665

Flow Cytometry:
Anti-CD3-BV421 (SK7), Biolegend, Catalog #: 344833 RRID: AB_2565674
Anti-CD134-PE (Ber-ACT35), Biolegend, Catalog #: 350003 RRID: AB_10641708
Anti-CD274-BV785 (29E.2A3), Biolegend, Catalog #: 329735 RRID: AB_2629581
Anti-CD137-APC (4-B4-1), Biolegend, Catalog #: 309809 RRID: AB_830671
Anti-CD4-FITC (RPA-T4), Biolegend, Catalog #: 300505 RRID: AB_314073

Validation

All antibodies used are publicly available through Biolegend. Biolegend provides the following statements on its website:

Antibodies used for co-stimulation:
Each lot of this antibody is quality control tested by immunofluorescent staining with flow cytometric analysis. FC - Quality tested IHC-F, Costim - Reported in the literature, not verified in house

Antibodies used for proteogenomics:
TotalSeq™ Antibodies
Bulk lots are tested by PCR and sequencing to confirm the oligonucleotide barcodes. They are also tested by flow cytometry to ensure the antibodies recognize the proper cell populations.
Bottled lots are tested by PCR and sequencing to confirm the oligonucleotide barcodes.

Antibodies used for flow cytometry:
Each lot of this antibody is quality control tested by immunofluorescent staining with flow cytometric analysis. FC - Quality tested

Flow Cytometry

Plots

Confirm that:

☒ The axis labels state the marker and fluorochrome used (e.g. CD4-FITC).

☒ The axis scales are clearly visible. Include numbers along axes only for bottom left plot of group (a 'group' is an analysis of identical markers).

☒ All plots are contour plots with outliers or pseudocolor plots.

☒ A numerical value for number of cells or percentage (with statistics) is provided.

Methodology

Sample preparation

PBMCs from 5 healthy donors were quickly thawed and placed in pre-warmed xVIVO15 cell culture medium (Lonza) supplemented with 5% heat-inactivated FBS. To reduce cell clumping, PBMCs were incubated in xVIVO15 containing 50 U/mL of benzonase nuclease (Sigma-Aldrich) for 15 minutes at 37 degrees and filtered using a 70 µm cell strainer. Washed and nuclease treated cells were seeded in a 96 well cell culture plate at a concentration of 2.5 x 106/mL. Peptide stimulations were performed using the CEFX Ultra SuperStim Pool (JPT Peptide Technologies, Product Code: PM-CEFX-1) at a final concentration of 1.25 µg/mL per peptide for 22 hours at 37 degrees and 5% CO2. Recombinant anti-CD28 and anti-CD49d antibodies (BioLegend) were added at a final concentration of 5 µg/mL and 0.625 µg/mL, respectively, to provide co-stimulation for peptide reactive T-cells. Separately mock-stimulated cells were treated with anti-CD28 and anti-CD49d antibodies at the same concentration. Peptide responsive T-cells were detected by the expression of the surface activation markers PD-L1, OX40, and CD137 via flow cytometry. Following the stimulation, peptide treated and mock-stimulated cells were washed in cell staining buffer (PBS + 2mM EDTA + 2% FBS) to end the stimulation. Fc receptor blocking was performed using a 1:50 dilution of Human TruStain FcX (Biolegend) in cell staining buffer for 10 minutes at 4 degrees. Cell viability staining was performed using a 1:500 dilution of Zombie Yellow Fixable Viability Dye (BioLegend) prepared in PBS for 30 minutes at 4 degrees. Surface staining was performed using 1:100 dilutions of BV421 conjugated anti-CD3, FITC conjugated anti-CD4, BV786 conjugated anti-PD-L1, PE conjugated anti-OX40, and APC conjugated anti-CD137 (BioLegend) for 25 minutes at 4 degrees in cell staining buffer. Following cell staining, antigen reactive and non-reactive T-cells were identified using a BD FACSAria II cell sorter and collected in cRPMI medium (100 U/mL penicillin-streptomycin + 2 mM L-glutamine + 10 mM HEPES + 0.1 mM non-essential amino acids + 1 mM sodium pyruvate + .05 mM 2-Mercaptoethanol) supplemented with 20% FBS.

Instrument

BD FACSAria II

Software

BD FACSDiva 9.4

Cell population abundance	CD4+ and CD3+CD4- cell populations were 58% and 25% of total live gated PBMCs in the peptide stimulated condition. Within peptide-stimulated CD4 T cells, 4.21% were AIM-positive and 81.7% were AIM-negative. Within peptide-stimulated CD8 T cells, 2.45% were AIM-positive and 74.9% were AIM-negative.
Gating strategy	Gating on CD3+CD4+ PBMCs isolated CD4 T cells. Gating on CD3+CD4- PBMCs isolated CD8 T cells. Gating on PDL1+OX40+ CD4 T cells was then performed to sort Antigen Induced Marker (AIM)-positive from AIM-negative cells. Gating on PDL1 +CD137+ CD8 T cells was performed to sort AIM-positive and AIM-negative cells.

☒ Tick this box to confirm that a figure exemplifying the gating strategy is provided in the Supplementary Information.
